# Optimizing training of time series diffusion models via similarity score functions: application to cyclic and acyclic motion with IMU data

**DOI:** 10.3389/frai.2025.1640948

**Published:** 2025-09-17

**Authors:** Heiko Oppel, Andreas Spilz, Michael Munz

**Affiliations:** AI for Sensor Data Analytics Research Group, Ulm University of Applied Sciences, Ulm, Germany

**Keywords:** diffusion model, time series, similarity score functions, synthetization, human activity recognition, sport climbing

## Abstract

**Introduction:**

Denoising diffusion probabilistic models have shown the capability to generate synthetic sensor signals. These models rely on a loss function that measures the difference between the noise added during the forward process and the noise predicted by the diffusion model, thereby enabling realistic data generation. However, the stochastic nature of the process and the loss function complicate the estimation of data quality.

**Methods:**

To address this issue, we evaluated multiple similarity metrics and adapted an existing metric to monitor both the training and data synthesis processes. The adapted metric was further fine-tuned on the input data to align with the requirements of a downstream classification task.

**Results:**

By incorporating the adapted metric, we significantly reduced the number of training epochs required without observing performance degradation in the classification task.

**Discussion:**

Our findings demonstrate that optimizing the training process using similarity metrics not only conserves computational resources but also shortens the training time for generative models, making them more efficient and practical for real-world applications.

## 1 Introduction

In machine learning classifier are used to identify pattern in samples to differentiate between multiple categories. Often the data basis is either missing samples from specific categories as it can be a time or cost consuming process or the data is of poor quality. In such cases Denoising Diffusion Probabilistic Models (DDPMs) have emerged as powerful generative tools to increase the sample space with meaningful representatives, for example in domains such as computer vision (Azizi et al., 2023) or time series (Rasul et al., 2021). Those samples are then used to achieve better results in the classification task. Therefore, the synthesized data has to increase the variation of the dataset while also retaining the main information from the activity.

The training of such a DDPM is based on the maximization of the log-likelihood, so, that the generated sample distribution matches the one from the real data (Ho et al., 2020). In order to achieve this, the loss function of the DDPM is defined as the mean squared error between the noise, that was estimated by the U-NET (Ronneberger et al., 2015) and the noise that was used in the forward process of the diffusion model. This ultimately guarantees the generation of synthetic data in the forward process.Though, it is not possible to estimate the quality of the generated data with this loss function or the resemblance to the real sequence. In image generation, to assess the quality of the generated images one can rely on human raters as Best-Rowden and Jain (2018) did. With time series data, this is not feasible. Among others, some studies rely on a qualitative analysis by using decomposition methods like t-SNE or analyzing the probability density functions (Naiman et al., 2024) between real and generated signals. The disadvantage of those approaches is their requirement for visual confirmation. It is not possible to reduce the similarity information to a single value. Another possibility is the usage of a discriminative score (Yoon et al., 2019). For this, a neural network is trained to differentiate between real and generated signals. Though, this is a time consuming process and depending on the dataset the network architecture has to be adapted. A study by Ramzan et al. (2024) used a Generative Adversarial Network (GAN) to generate synthetic sequences from the domain of finance. They evaluated their synthetic sequences by using four different metrics: Kullback-Leibler (KL) Divergence, Wasserstein Distance, Energy Distance and the Maximum Mean Distance. Those are measures between probability distributions. Unfortunately it is not clear what distributions they compared. It is questionable to use those metrics in the time domain on time series signals as a comparison metric. They do not take into account the temporal progression of the signals. Narteni et al. (2025) also relied on GANs to generated synthetic sequences. Though, they investigated a rule-based classifier as evaluation metric for the synthetic data. A Logic Learning Machine was used to generate the set of rules automatically once for the real and once for the augmented data. Afterwards the similarity between the set of rules was compared. The Context Fréchet Inception Distance (FID) (Jeha et al., 2022) is another approach that relies on the usage of a neural network model. In this case, the TS2Vec (Yue et al., 2022) model is used. It is able to map each time step of a time series to a contextual representation by learning a non-linear embedding function. Some studies did also rely on similarity score functions to estimate the similarity between real and generated signals. So did Liao et al. (2023) by calculating the absolute error of the auto-correlation estimator. Suh et al. (2024) applied a similar methodology, but used the pair-wise column correlations among other evaluation methods. Finally, some studies use an underlying classification or regression task to objectively estimate the quality of the generated signals (Yoon et al., 2019; Suh et al., 2024; Oppel and Munz, 2025). They evaluate the separability of the classifier with and without the addition of synthetic data.

To sum it up, in the literature, there exist several approaches to evaluate the quality of the generated data, though, they were either not used to monitor the training progress of a diffusion model or are not suitable to do it. Therefore, we introduce a similarity score novel to the domain of time series comparison analysis, and also integrate it in the training and denoising process of a time series diffusion model by estimating the models ability to generate comparable signals. To achieve this we developed a method called Class Optimization Global Alignment Kernel (C-Opt GAK) to optimize the similarity score's power of describing the dataset. We do this by first calculating the power spectral density (PSD) of the signals and then estimate the similarity scores fit based on a preceding signal analysis. We compared the optimization process against several other subjectives to show the robustness of our approach and evaluated the metric itself against other established time series metrics. Those were the root mean squared error (RMSE), the Pearson correlation coefficient and the cosine similarity.

The second contribution of this work is the integration of similarity metrics in the training and denoising process of a generative diffusion model to control its process, speed up training while improving or maintaining the data quality. To the best of our knowledge, this is the first time that time series similarity score functions are used for monitoring the training and denoising progress of a diffusion model. So far, monitoring the training progress was done by relying on the loss value alone.

This work has the following main contributions:

- We propose a similarity score function new to the domain of evaluating synthetically generated time series signals and use an optimization process to best fit it to the real data.
- We integrate similarity score functions in the training progress of a DDPM to reduce the amount of training epochs whilst maintaining or even improving the quality of the generated sequences.
- We use the similarity score functions to reduce the amount of denoising steps without decreasing the quality of the generated signals for the underlying classification task.

This paper is structured as follows. At first, the methods section introduces the utilized datasets including the processing stages necessary to reproduce the results. We then provide information about the similarity metrics and how to apply them to the underlying task. The results section is divided into four parts. At first, we examine the benefits of using similarity scores for monitoring the training process of a diffusion model, then we analyze the monitoring of the denoising process before highlighting the classification results. The last part builds upon the findings and evaluates the results obtained by applying the approach to an acyclic movement dataset.

## 2 Methods

This is a follow-up study based on the work of Oppel and Munz (2025). The processing of the data, the choice of the classes, the DDPM model (IMUDiffusion) and classifier configuration are explained in detail in this study. For more information, please refer to the original publication.

### 2.1 The datasets

We tested the developed approach with two different datasets. The first is about cyclic human movements from a human activity recognition dataset, denoted as HAR dataset in the following. The second is about tracking climbing movements with an instrumented belay device, denoted as “climbing dataset” in the following. Compared to the HAR dataset, the latter one does not hold cyclic movements as each fall of a climber into the rope, as well as each movement on the wall provides a timely limited movement behavior of the belay device. If not otherwise stated, the evaluation and analysis is performed on the human activity recognition dataset.

#### 2.1.1 Human activity recognition dataset

For this study, we used a HAR dataset on the basis of Inertial Measurement Units (IMUs) introduced by Baños et al. (2012). The original aim was to analyze the effect of IMU displacement. They recorded 17 participants performing 33 activities from which we chose four: Walking, Running, Jump Up and Cycling. As some of the participants did not participate in all activities, we reduce the pool of participants to those 12 which performed all activities. For a better readability when addressing single participants, we will further address them as PID *x* (participant with the id *x*, *x*∈1, ..., 16). Furthermore, we only used the IMU located on the right thigh with the ideal placement setup. This guarantees comparable movement pattern along the IMU axes.

#### 2.1.2 Climbing dataset

The climbing dataset was initially recorded by Oppel and Munz (2022). See the original publication for more details on the study protocol. In total, over 150 climbing falls were recorded altogether with over 60 ascents from different climbers including varying climbing scenarios. or this paper, we reduced the dataset to 37 climbing falls and 19 ascents. This is leading to an intentionally imbalanced dataset of 1:9. The ascents and falls were recorded using the same belay device and electronic hardware. The climbing falls can be further divided into five different configurations depending on the amout of slack (loose rope), and the fall potential. Fall potential means the height of the climber above the last anchor. Both parameters influence the fall distance and the dynamics of the fall. The five confiurations were: no slack, fall potential of 0*m*, 0.25*m* and 0.5*m*, and fall potiential of 0*m*, with slack of 0.5*m* and 1*m*. The belay device was held firmly in the hands of the belayer and the breaking mechanism of the belay device was deactivated to guarantee a fall of at least two meter. For each of those falls we used a sandbag as a substitute for the climber to not endanger a human climber. To the sandbag, a timely synchronized IMU was attached to extract the required label information of the fall itself. The climbing ascent recordings can be further divided in six categories including the clipping position of the climber (stretched out or around the thorax), slack in the system (no slack or ≈1.5*m* of slack) and the type of belaying (active or passive).

In order to record this dataset, we integrated an IMU and three bipolar Hall-Sensors into a belay device to record its the movement behavior while climbing.

### 2.2 Signal processing

The two evaluated datasets require different pre-processing steps which are addressed in this section as well as some common processing steps across the two datasets.

#### 2.2.1 Pre-processing steps for the HAR dataset

The HAR activities were recorded with a sample rate of 50*Hz*. We further sequenced the data with a sliding window width of 160 time steps and an overlap of 40 time stemps. Those signals are transformed into the frequency domain using a short time fourier transform (STFT) using a window size of 22 and an overlap of 20. Windowing has been done using the Hanning function. The frequency domain signal is then used as input into the diffusion model.

#### 2.2.2 Pre-processing steps for the climbing dataset

The climbing dataset was recorded with a sample rate of 220 *Hz* for each sensor type: accelerometer, gyrometer and Hall-Sensor state. Using the data from the accelerometer and gyrometer, we rotated the IMU from its local coordinate system to the geocoordinate system using an AHRS algorithm (Madgwick et al., 2011). Afterwards, the data was separated using the information from the sandbag or climber to split it into three different classes: Falling, Rope-Pull and Stillstanding. The two classes Rope-Pull and Stillstanding were both extracted from the ascents and hold different kinds of information. Rope-Pull includes only sequences, where rope movement was registered in the belay device. Additionally, we added 20 more samples before the initial registration of rope movement to include the movement of the belay device, as the belay device is typically being moved before rope is handed out. This reduces the Stillstanding class to moments where no movement was registered in the belay device or movements due to active belaying in the moment before rope was handed out. The start time of the fall sequences was chosen to be identical to the sequences of the Rope-Pull class. In the next step each sequence was set to 160 time steps and then transformed to the frequency domain using STFT in the same way as the HAR dataset. The signal was then normalized before beginning the training of the DDPM.

#### 2.2.3 Power spectral density

The main goal of synthesizing data is to add variation to the dataset while retaining the main information from the activity. Comparing sequences in the time domain may either suggest to use sequences that are highly similar, hence, not increasing the variance within the dataset or, even worse, it can lead to the assumption, that sequences are fairly dissimilar while having the key information of the activity, yet, deviate from the real sequences. To address this issue, we estimate the signals' power spectral density (PSD) using Welch's method (Welch, 1967). This method estimates the PSD by first separating the signal into *K* windowed subsequences


(1) $$\begin{array}{l} x_{\omega ,k}=\omega x_{k}\text{, with k=0,1,...K-1} \end{array}$$

where ω represents the window function. For each subsequence, the periodogram *P*_*x*_ω, *k*_, *M*_(ω) is then calculated


(2) $$\begin{array}{l} P_{x_{\omega ,k},M}\left(\omega \right)=\frac{1}{M}|\overset{M-1}{\underset{m=0}{\sum }}x_{\omega ,k}\left(m\right)\cdot e^{-j2\pi m/M}{|}^{2} \end{array}$$

where *M* denotes the sequence length of each subsequence. Finally, by taking the average over all periodograms we get the power spectral density


(3) $$\begin{array}{l} {Ŝ}_{x}\left(\omega \right)=\frac{1}{K}\overset{K-1}{\underset{k=0}{\sum }}P_{x_{\omega ,k},M}\left(\omega \right) \end{array}$$

Using this approach removes the temporal dependency in the course of the sequence. The idea behind this is to focus on the main characteristics in the signal that represents the activity independent of the location in time.

### 2.3 Similarity metrics

#### 2.3.1 Class-optimized global alignment kernel

Global Alignment Kernel (GAK) *k*(*x, y*) is an approach to map a sequence *x* onto another sequence *y*. As stated in Cuturi (2011), it exponentiates the soft-minimum of all alignment distances and is defined as


(4) $$\begin{array}{l} k\left(x,y\right)=\underset{\pi \in A\left(n,n\right)}{\sum }e^{-D_{x,y}\left(\pi \right)}\text{,} \end{array}$$

whereas π being an alignment path, A(n,n) the set of all alignments between the two sequences *x* and *y* of length *n* and *D* is the cost of the alignment π. An alignment path is a sequence of index pairs which best map the sequences *x* and *y* onto each other. The cost *D* is defined by Equation 5 and its exponentiation bounds each element to [0, 1].


(5) $$\begin{array}{r} D_{x,y}=d\left(x,y\right)-\ln(2-\exp\left(d\left(x,y\right)\right)\text{, with}d\left(x,y\right)=-\frac{\varphi \left(x,y\right)}{2\sigma^{2}}\\ \text{and}\varphi \left(x,y\right)=\sqrt{{\left(x-y\right)}^{2}} \end{array}$$

**Figure 1:**
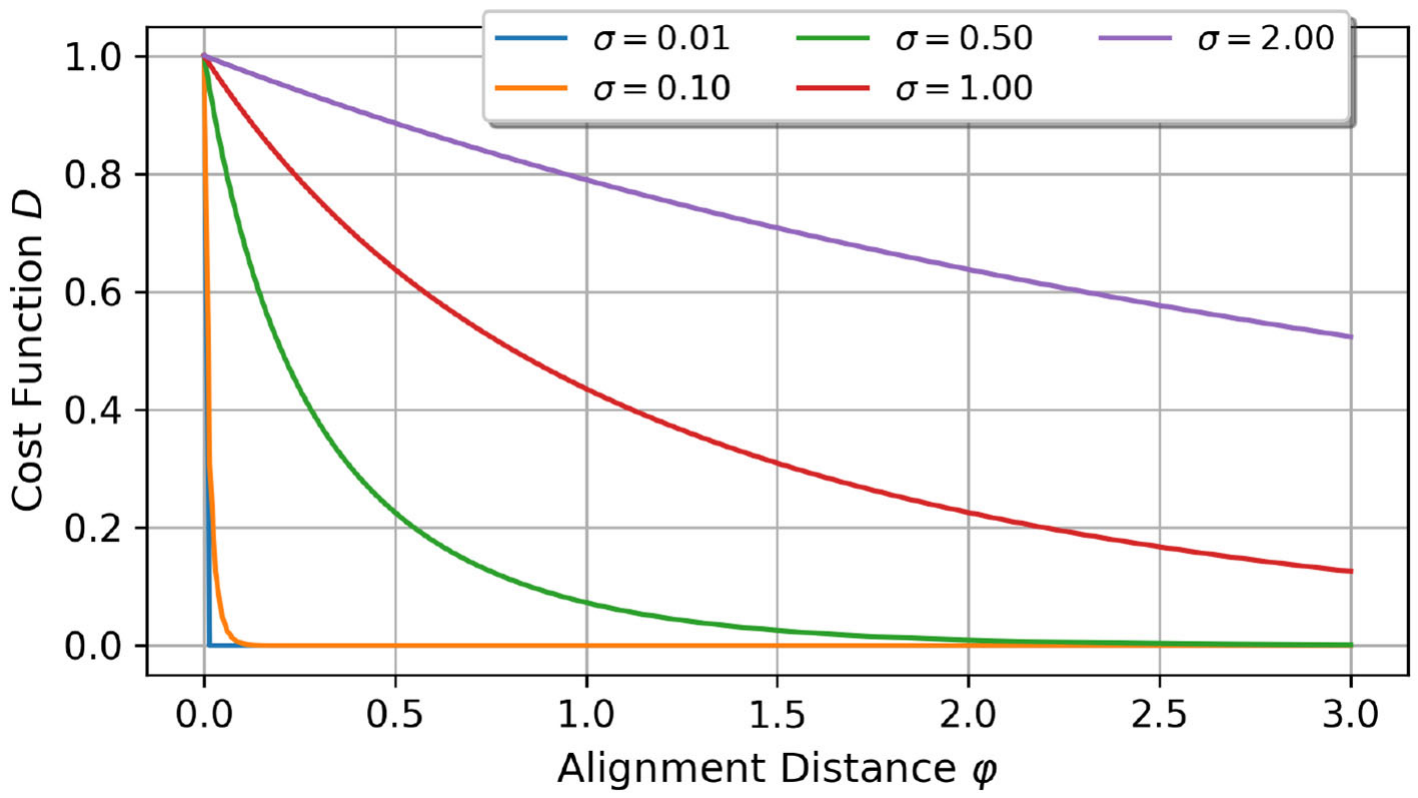
Comparison of different scaling factors σ and their impact on the cost function *D* over the alignment distance. For lower scaling factors, the cost function is more sensitive to smaller alignment distance changes.

Each operation in calculating the cost function is an element-wise operation. The scaling factor σ is responsible for the scaling of the distance function, and, hence, on the cost function *D*, see Figure 1. In summary, by increasing σ, the cost function approaches its limit value 0 slower.

Finally, we normalize the global alignment kernel *k*(*x, y*) according to Equation 6.


(6) $$\begin{array}{l} \vartheta \left(x,y\right)=\frac{k\left(x,y\right)}{\sqrt{k\left(x,x\right)\cdot k\left(y,y\right)}}\in \mathbb{R}:\vartheta \left(x,y\right)\in \left[0,1\right] \end{array}$$

##### 2.3.1.1 Estimation of the optimal σ-value

The GAK is directly dependent on the scaling factor σ∈ℝ:σ>0. It is a sensitive parameter responsible for the degree of selectivity of the similarity between two sequences. A high degree of selectivity means that small variations in the data are able to change the value of the GAK significantly. The lower the value, the higher the degree of selectivity. Cuturi (2011) suggest to calculate the scaling factor based on the median distance between various timesteps across the two time series and scale it. It is even possible to use a multiple of the scaled median distance. We evaluated their approach by calculating the GAK between sequences from our training and validation set, which should have a high degree of similarity. Though, it lead to an average σ-value of 7.15·10^−4^±4.87·10^−4^, equivalent to a high degree of selectivity, and hence, made it not usable for our concept. Therefore, we change the approach of estimating the optimal scaling factor. As previously mentioned, we assume a high similarity between sequences from the training and validation set. So, we perform an optimization by calculating the maximum of the average GAK value across all sequence pairs under the condition, that the standard deviation is in the range [0.09, 0.12]. Due to the cyclic behavior of the activities, we assume a high similarity between data in the training and validation sets. Therefore we analyzed the similarity between those sets in combination with the similarity score and finally decided on the previous mentioned range. As this is a subjective assessment, it requires knowledge about the underlying dataset. The mathematical formulation is as follows:


(7) $$\begin{array}{r} \begin{array}{r} C_{GAK}=\max\left(\bar{\vartheta }\left(x,y\right)\right),\\ \text{subject to }{\hat{\sigma }}_{\vartheta }\in \left[0.09,0.12\right], \end{array} \end{array}$$

with $\bar{\vartheta }\left(x,y\right)$ and ${\hat{\sigma }}_{\vartheta }$ being the average and standard deviation of the GAK values. This adapted GAK metric will be further referenced as the class optimized global alignment kernel as introduced in the introduction (or short: C-Opt GAK).

**Figure 2:**
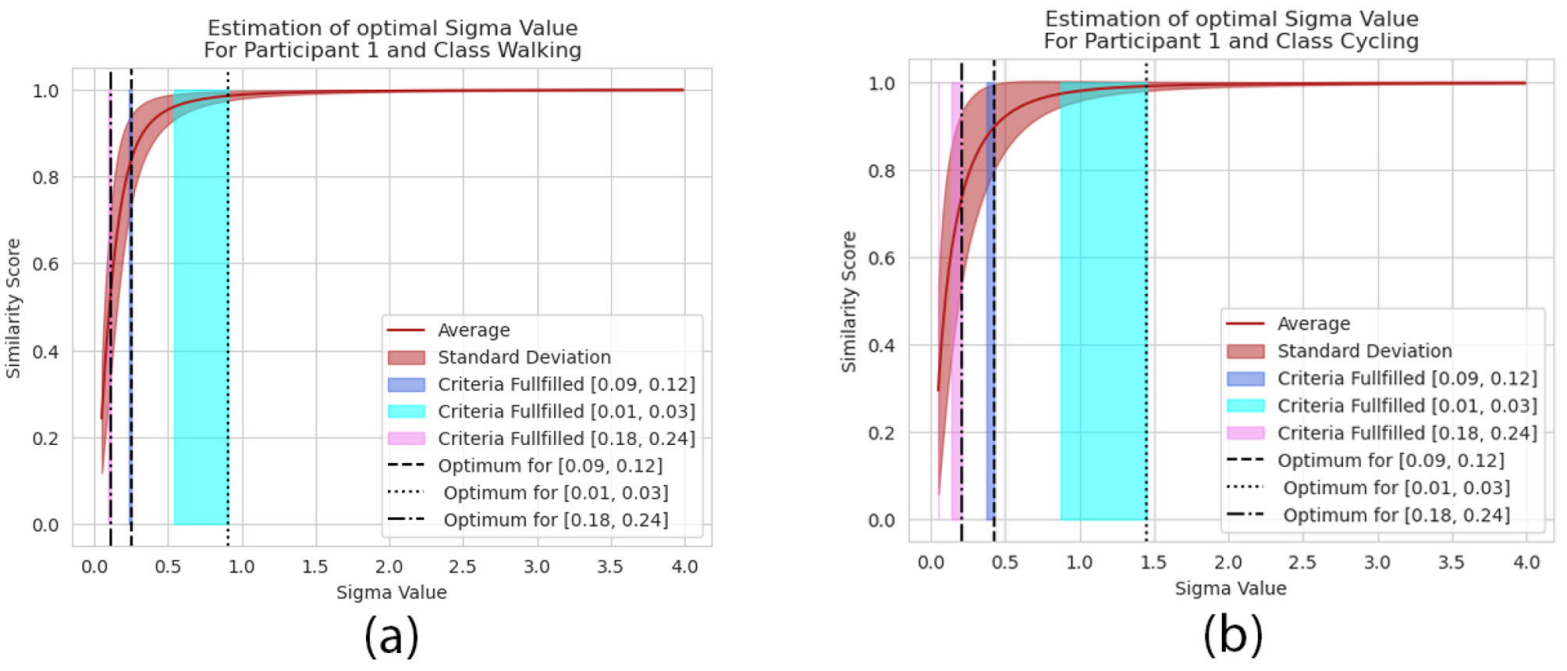
Visual representation of the σ-value and its influence on the similarity score for a given train and validation set from the HAR dataset. Highlighted are the different pre-defined ranges ${\hat{\sigma }}_{\vartheta_{i}},\forall i=1,2,3$. They are represented by the colored areas in blue, cyan and pink. Those are the areas where the objective constraint from Equation 7 are met and the dotted lines represent the optimal σ-value according to Equation 7. It is exemplarily visualized for for the Cycling activity performed by the participant ID 1 **(a)** and for the Walking activity performed by the participant ID 1 **(b)**.

A visual representation of the identification of the optimal σ-value is presented in Figure 2. It visualizes the similarity score over a range of σ-values. The dark blue range defines the area in which the criteria according to Equation 7 is fulfilled. The red curves and their slope describe the average and standard deviation of the most similar sequences. Depending on the input sequences, the σ-value is able to change the interpretation of the GAK value, compare the Figures 2a, b.

**Figure 3:**
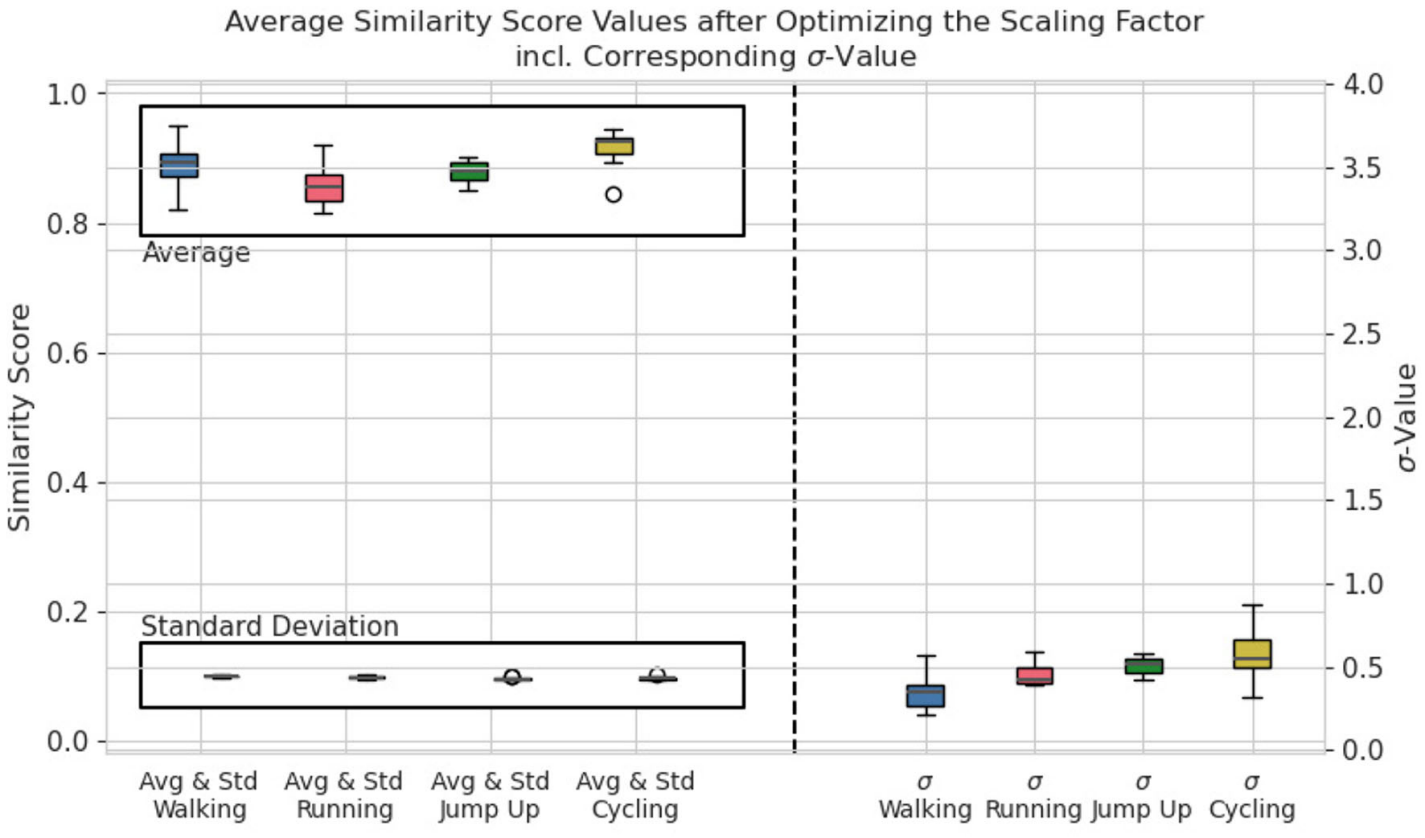
Boxplot of the average and standard deviation of all similarity scores **(left side of the graph)** and of the scaling factors σ **(right side of the graph)** of all participants grouped by activity for ${\hat{\sigma }}_{\vartheta_{2}}=\left[0.09,0.12\right]$.

A summary of all calculated σ-values of all participants is presented in Figure 3 for ${\hat{\sigma }}_{\vartheta_{2}}=\left[0.09,0.12\right]$. They range from 0.1 in case of the Walking class up to almost 1.0 for participants performing the Cycling activity. Each class requires a specific range of σ-values to meet the criteria defined in Equation 7. In addition to the σ-values, Figure 3 visualizes the average and standard deviations of the C-Opt GAK values between the training and validation set. It is further separated by the individual classes. The standard deviations ${\hat{\sigma }}_{\vartheta }$ show little variations as they are strictly limited by the condition. The dispersion on the mean values $\bar{\vartheta }$ on the other hand differ between the activities.

#### 2.3.2 Impact and evaluation of different optimization constraints

As previously mentioned, the range of the standard deviation ${\hat{\sigma }}_{\vartheta }$ is a subjective procedure and depends on the dataset. We compared our chosen range in the optimization process against two other optimization constraints ${\hat{\sigma }}_{\vartheta }$ and examined their impact on the classification task and analyzed their plausibility against each other. The three ranges are as follows:

-
${\hat{\sigma }}_{\vartheta_{1}}=\left[0.01,0.03\right]$

-
${\hat{\sigma }}_{\vartheta_{2}}=\left[0.09,0.12\right]$

-
${\hat{\sigma }}_{\vartheta_{3}}=\left[0.18,0.22\right]$

**Figure 4:**
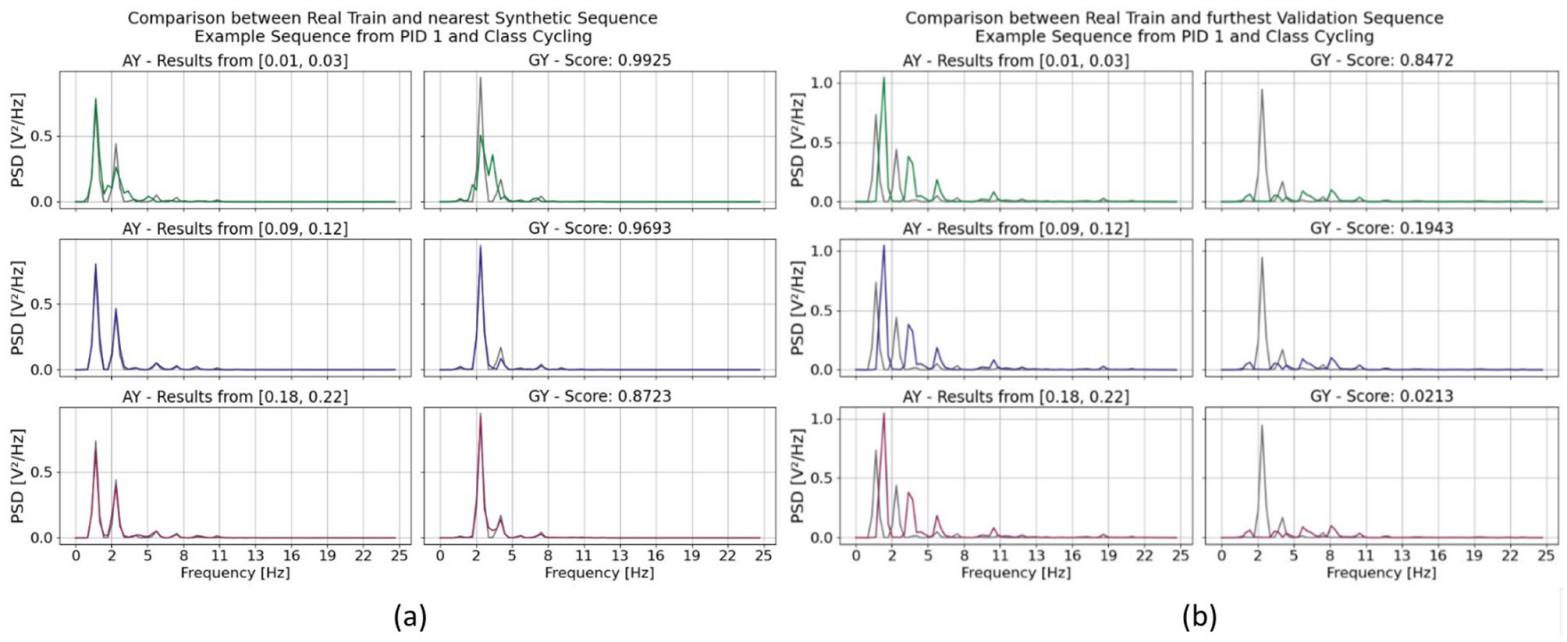
Exemplary comparison between two sequences from the HAR dataset. **(a)** Between a real training sequence and a synthetic sequence and **(b)** between two real sequences, one from the training and one from the validation set. It can be further separated by the three optimization ranges ${\hat{\sigma }}_{\vartheta_{i}}\forall i=1,2,3$ of the standard deviation to estimate the scaling factor σ. They are color coded in the following way: (green) ${\hat{\sigma }}_{\vartheta_{1}}=\left[0.01,0.03\right]$, (blue) ${\hat{\sigma }}_{\vartheta_{2}}=\left[0.09,0.12\right]$ and (red) ${\hat{\sigma }}_{\vartheta_{3}}=\left[0.18,0.22\right]$. The C-Opt GAK similarity score value is also given for each sequence comparison on average across all channel in **(a, b)**. In those subgraphs, only two of the six channel are visualized, namely the acceleration in y-direction (AY) and angular velocity in y-direction (GY).

They are exemplarily visualized in Figures 2a, b. The visual analysis shows that the range directly impacts the interpretations of the similarity between two signals. For a more detailed analysis on their impact see Figure 4. There we compare two sequences (their PSDs) against each other for each range separately. At first, we were interested in the two sequences which are resembling each other the most. So, we calculated the similarity scores between a randomly pre-chosen real sequence and all available synthetic sequences. Then we selected that synthetic sequence which returned the highest similarity score. The results are visualized in Figure 4a. The two ranges ${\hat{\sigma }}_{\vartheta_{2}}$ and ${\hat{\sigma }}_{\vartheta_{3}}$ ultimately lead to sequences which visually confirm a high similarity between them. Though, their score value differs. As ${\hat{\sigma }}_{\vartheta_{2}}$ returns a plausible score value 0.9693, ${\hat{\sigma }}_{\vartheta_{3}}$ returns with 0.872 a lower value. The highest score value was calculated by when the range ${\hat{\sigma }}_{\vartheta_{1}}$ was used. It lead to a value of 0.9925 which indicates a high similarity between the two sequences. Though, their similarity is clearly less pronounced.

In a second approach, we analyzed the two least similar sequence according the similarity score and compared the impact of the three standard deviation ranges against other. The exemplary result is shown in Figure 4b. The score value of the least similar sequence was identical for all ranges. Though, again, the value of the C-Opt GAK score varied between 0.021 and 0.847 which exhibits either a high similarity or no similarity at all. By visually analyzing the sequences, it gets clear, that a high score value of 0.847 is not a plausible value. The remaining two score values of 0.021 and 0.194 are both plausible, as the value depends on the application itself.

#### 2.3.3 Comparison similarity metrics

We choose three time series similarity metrics to evaluate our C-Opt GAK metric against—the Cosine similarity, the Pearson Correlation Coefficient and the Root Mean Squared Error (RMSE). Each metric is used to calculate the similarity scores in the time domain in addition to analyzing the similarities of the signals power spectral densities.

The Cosine similarity *s*_*c*_ between two sequences *x* = (*x*_1_, ..., *x*_*n*_) and *y* = (*y*_1_, ..., *y*_*n*_) is calculated by taking the dot product between two sequences and additionally norming it using their magnitudes ||*x*|| and ||*y*||:


(8) $$\begin{array}{l} s_{c}\left(x,y\right)=\frac{xy}{\left||x||\cdot ||y|\right|}. \end{array}$$

The second similarity metric is the Pearson Correlation Coefficient *s*_*p*_:


(9) $$\begin{array}{l} s_{p}\left(x,y\right)=\frac{n\sum_{i}x_{i}y_{i}-\left(\sum_{i}x_{i}\cdot \sum_{i}y_{i}\right)}{\sqrt{\left(n\sum_{i}x_{i}^{2}-{\left(\sum_{i}x_{i}\right)}^{2}\right)\cdot \left(n\sum_{i}y_{i}^{2}-{\left(\sum_{i}y_{i}\right)}^{2}\right)}},\forall 1<i<n \end{array}$$

Finally, the RMSE is calculated as follows:


(10) $$\begin{array}{l} s_{r}\left(x,y\right)=\sqrt{\frac{\sum_{i}{\left(x_{i}-y_{i}\right)}^{2}}{n}} \end{array}$$

Both Cosine similarity and Pearson Correlation Coefficient are in the range of [−1, 1], the score value calculated by the RMSE is in the range of ℝ.

#### 2.3.4 Visual analysis of exemplary sequences between the similarity metrics

**Figure 5:**
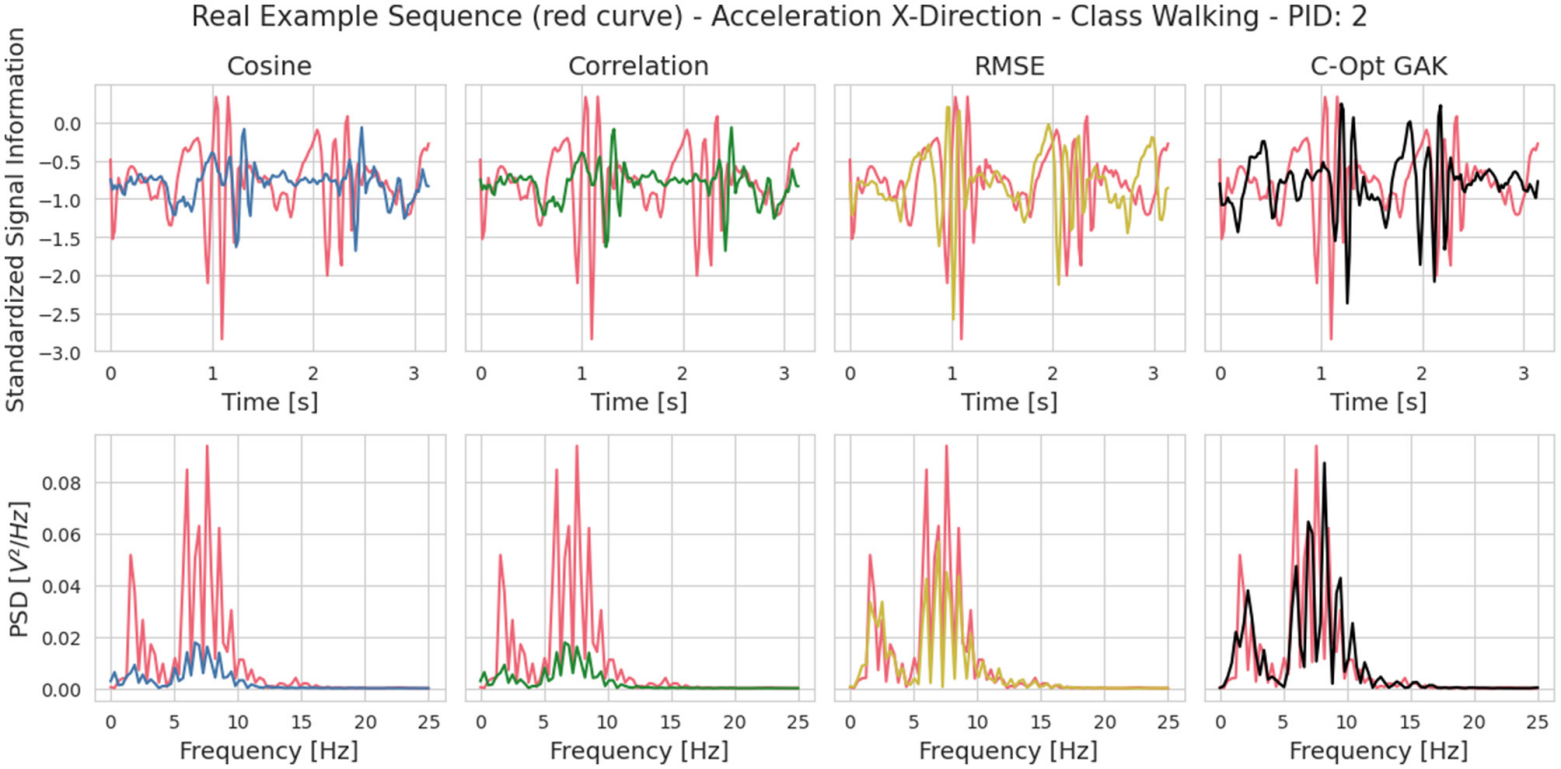
Visual comparison between a sequence from the training and validation set. The sequence from the training set was randomly chosen and the sequence from validation set was chosen based on the similarity metric. It is the sequence that resembles the training sequence the most according to the respective similarity metric. The sequence from the training set is visualized in red. The respective sequence from the validation set is visualized in a different color depending on the similarity metric that led to choosing the respective sequence (blue: Cosine, green: Correlation, yellow: RMSE, black: C-Opt GAK). The **top row** visualizes the sequences in the time domain and the **bottom row** their respective power spectral density.

Figure 5 visualizes an example sequence showing the acceleration in x-direction from PID 2 of the Walking class once in the time domain and once its power spectral density (red curve in the figures). We have visually compared this sequence with the most similar sequence from the validation set according to all four metrics. To determine the most similar sequence, we calculated the similarity scores individually across all sensor axes and then averaged them. The calculation was done between the power spectral densities of the sequences. The Cosine and Correlation metric chose identical sequences, whereas the RMSE and C-Opt GAK approaches chose different sequences. The choice for identical sequences between the Cosine and Correlation metric was observed across all classes. We have therefore decided not to consider the Correlation metric further in our analysis. One of the disadvantages of using RMSE as a similarity score is the lack of interpretability of the score value itself. The only assumption that can be made is the following: the lower the score value, the higher the similarity between two sequences. Visually, both the sequences chosen by the RMSE as well as the C-Opt GAK show high similarities toward the sequence from the training set. Therefore, we also excluded the RMSE score in our further analysis.

### 2.4 Denoising diffusion probabilistic model

The IMUDiffusion model is a diffusion model specifically designed for synthesizing time series sequences based on multiaxial IMUs. It was first introduced by Oppel and Munz (2025) which showed the effectiveness of the generated sequences by improving the underlying classification task of separating human motion activities. The model description can be found in their paper. They trained the diffusion model for 4, 500 epochs, which will be the reference for this study.

For the noise scheduler, a linear scheduler was applied. It was adapted to the multi-sensor problem by choosing separate diffusion rates per sensor.

#### 2.4.1 Application to the climbing dataset

The scheduler beta values for the Accelerometer and Gyrometer were identical to the ones when training the DDPM on the HAR dataset, and the rope velocities beta value was set to β_*velo*_ = 9*e*−4.

The DDPM was trained on each recording separately to guarantee high similarities between the synthetically generated and the real ones. A recording comprises of a single sequence in the case of the fall sequences and multiple sequences for the Rope-Pull and Stillstanding class. The number of samples in the ascent recordings varied according to the amount of rope pull registrations and comprised of up to 27 sequences.

### 2.5 Classifier

Like the diffusion model, we rely on the same classifier architecture introduced by Oppel and Munz (2025). This allows for a comparability between the results to analyze the effectiveness of the similarity score. The classifier is composed of a convolutional neural network that convolves the input only along the time dimension. We use two convolutional layers with a kernel of size *c*_*kernel*_ = (1 × 5) and 2 filter each, followed by a Max-Pooling layer to reduce the dimensionality along the time by two. After the Max-Pooling layer an additional convolutional layer with 4 filter and the same kernel size as the previous convolutional layer was used. Finally, the last part of the network consists of three fully connected layers with 128, 32 and 16 neurons respectively. Each fully connected layer is followed by a ReLU activation layer and a dropout layer with *p* = 0.3. The last layer of the network consists of four neurons followed by a softmax layer. The same classifier model was used for both datasets.

### 2.6 Experiments

The similarity metrics Cosine Similarity and the C-Opt GAK are used to monitor the training and the denoising process of the DDPM. Both metrics are used to compare the power spectral densities of the sequences. Additionally, the Cosine similarity was also used to compare sequences in the time domain.

In each experiment, we trained the DDPM and the classifier using the Leave-one–subject-out Cross Validation (LOSOCV) method. Each participant was once excluded from the training and validation set and only used for testing. As we have 12 participants in total, we trained 12 classifier models and evaluated the results separately. The same methodology has been used to train the DDPM. Though, the training of the DDPM was additionally separated by the classes to guarantee a unique label for the synthetic sequences. This results into a total of 48 DDPMs.

#### 2.6.1 Monitoring the DDPM training

By monitoring the training progress of the DDPM using the similarity score functions, we are able to estimate the quality of the synthetic sequences at any desired epoch. To do this, we are denoising a batch of 128 randomly normal distributed sequences in the frequency domain for the full 3, 000 denoising steps after the specific epochs. Though, as this is a time consuming process, we reduce the amount of monitored epochs to every 50^*th*^. Now, the termination criteria between the C-Opt GAK and Cosine metrics vary. Using the Cosine metric for monitoring the training process, we search for a local maxima of this metric between real training sequences and the batch of synthetic sequences. Additionally, as the score value can be volatile, we keep training for another 100 epochs including two monitoring steps, to ensure that an optimum has been reached. The C-Opt GAK method allows us to be more specific with the criteria for terminating the training process. By optimizing the scaling factor σ using the real training and validation sets, we also estimate the range of the similarity score. Therefore, we expect the similarity score between real training sequences and synthetic sequences to be in the same range. In practical terms, we require that at least 25% of the similarity scores to be in the range. If both criteria were met, we stop the training process.

#### 2.6.2 Monitoring the denoising process

The scheduler is responsible for controlling the denoising process. Initially, we set the number of denoising steps to 3, 000. With the help of the similarity scores, we are able to monitor this process and estimate the quality of the synthetic sequences by comparing them against the real training sequences. We used the information of the similarity scores to stop the denoising process as soon as the optimal quality of the synthetic sequences was reached. Again, we allow two additional monitoring steps to guarantee that the local optimum was reached. Therefore, if in two consecutive steps the similarity score drop, we stop the denoising process. Again, this is a very time consuming process if every denoising step is monitored. Therefore, we monitored only every 30^*th*^ step.

#### 2.6.3 Training sets for the HAR classification task

In order to objectively evaluate the quality of the synthetic sequences, we add those sequences to the training set for classifying the four activities Walking, Running, Jump Up and Cycling. Overall, we compare 9 training sets against each other that have been used to train a neural network classifier with identical architecture and initial weights. First of all, we have the two baseline sets—namely the “Full-Set” and the “2 Sample Set.” The Full-Set comprises 80% of the available data from 11 participants. The remaining 20% from those participants are used for validating the classifiers performance. Finally, the left-out participant was used for testing. Therefore, the test set was always identical, independent of the training sets. In case of the 2 Sample Set, the training data comprises 2 randomly chosen samples out of the real samples per participant, leading to a total of 22 real samples in the set. The same amount but different samples, were chosen for the validation set. The final baseline set is the Full DDPM Set. It consists of synthetic sequences which have been generated with the IMUDiffusion model without the usage of similarity metrics to monitor the training and synthetization process. Meaning, the IMUDiffusion model has been trained for 4, 500 epochs and the sequences have been denoised for 3, 000 steps. The same real sequences from the 2 Sample baseline set were used to train the diffusion models.

The results obtained by the classifier with the baseline sets serve as a reference against the results obtained from training the classifier with a different training set that contains synthetic sequences which have been generated with the help of the similarity metrics. Those two metrics were the C-Opt GAK and Cosine similarity and were either applied to monitor the training of the IMUDiffusion model or its denoising process. Depending on the similarity metric, each have generated different synthetic samples which have been separately used for training the classifier. An additional control parameter is the application of the similarity score either directly onto the time signals or onto their power spectral densities. A summary of all variants are shown in Table 1. In total, classification results from 9 different training sets have been evaluated. For further simplifications, we use the preceding abbreviation “OT” (Optimal Control Training) for the training set which contains synthetic sequences that have been generated with the IMUDiffusion model according to Section 2.6.1. If additionally the denoising process has been monitored, the abbreviation “OT-D” (Optimal Control Training with Denoising) is applied.

**Table 1:** Summary of the HAR training sets that were used to the train the classifier.

**Training set name**	**Number of real training samples**	**Synthetic sequences added**	**Number of training epochs**	**Number of denoising steps**	**Similarity metric**	**Similarity measured between**
2 Sample	22	None	4,500	3,000	None	None
Full-set	≈ 25,000	None	4,500	3,000	None	None
Full DDPM	22	15,360	4,500	3,000	None	None
OT C-Opt GAK	22	15,360	Optimal control	3,000	C-Opt GAK	PSD
OT-D C-Opt GAK	22	15,360	Optimal control	Optimal control	C-Opt GAK	PSD
OT cosine PSD	22	15,360	Optimal control	3,000	Cosine	PSD
OT-D cosine PSD	22	15,360	Optimal control	Optimal control	Cosine	PSD
OT cosine time	22	15,360	Optimal control	3,000	Cosine	Time
OT-D cosine time	22	15,360	Optimal control	Optimal control	Cosine	Time

Included are several key factors that describe how the sets were constructed.

#### 2.6.4 Training sets for the climbing classification task

The amount of recordings varies between the fall and ascent classes. Therefore it was not possible to perform a Leave-One-Recording-Out Cross-Validation. Therefore, we split the dataset five times. The split was performed on the recordings. This guaranteed that no handing out rope sequence of the same recording/ascent was present in both the training and the test set.

In a previous study by Oppel and Munz (2024) they analyzed different time window sizes for predicting a climbers fall into the rope and found a well balanced compromise in a window size of 20 time steps. For the synthetisation process we already reduced the sequences to a window size of 160 time steps. Those were then further processed by using a new window size of 20 time steps with a slide of 10. After the sequencing of the data, the accelerations and angular velocities were standardized and the rope velocity was normalized as the data distribution is not normal and due to outlier velocities in some fall situations.

In order to analyse the impact of the similarity score on the prediction of climbing events by using synthetic sequences generated with a DDPM, we evaluated six datasets:

- Train-On-Real-Test-On-Real (TRTR): using all available Sequences for training the classifier without the held out test set.
- TRTR with Downsampling: due to the class imbalance of the dataset, we sampled the majority classes from the climbing ascent down to match them with the amount of fall sequences.
- TRTR with Oversampling: we increased the sample space by replicating the fall sequences until we reached a class balance.
- Last Step Train-On-Synthetic-Test-On-Real (TSTR): we used the DDPM model after 4,500 epochs of training to synthesize climbing sequences which were then used for training the classifier without any real sequences in the training process.
- Best Step TSTR: we used the DDPM model at the epoch where our similarity score estimated an optimal similarity between the synthetic and real training sequences and used those synthetic sequences alone to train the classifier.

## 3 Results

The results section is divided in three parts. The first two parts analyse the findings from integrating the similarity scores in the training and synthetization process of a diffusion model. In the last part, the results of using synthetic sequences for training a classification model are discussed.

### 3.1 Monitoring the DDPM training process

**Figure 6:**
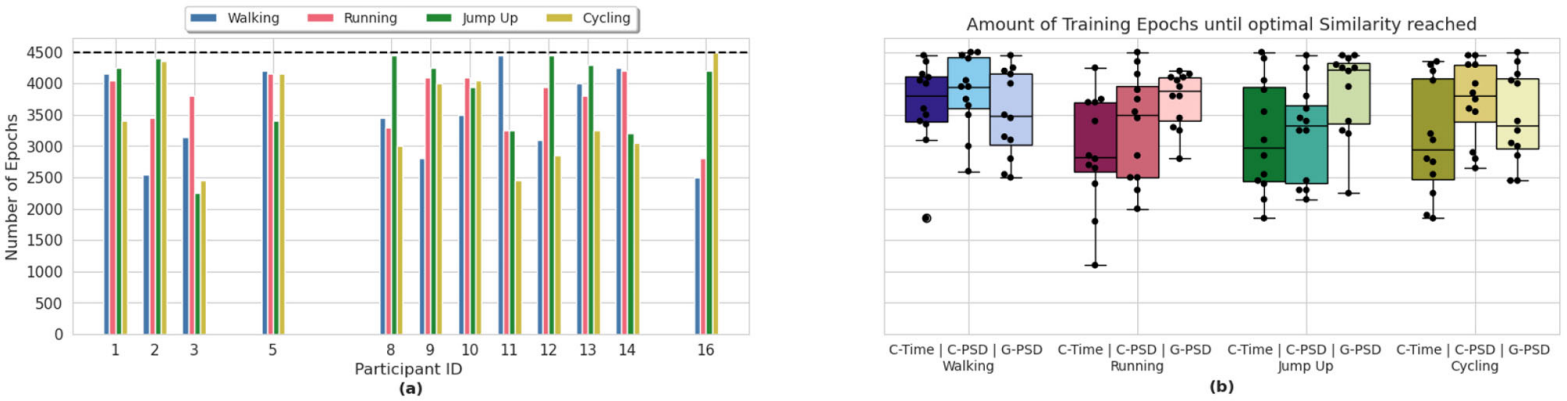
Number of training epochs until the similarity score induced an early stopping of the training process. **(a)** visualizes the participant and class individual result from the HAR dataset when using the C-Opt GAK similarity metric for early stopping. The dashed black line represents the pre-set amount of training epochs. **(b)** swarm-box-plot of the results of all evaluated similarity metrics, grouped by the classes. Each black dot in the swarm-plot represents one participant.

We have integrated the similarity scores in the training process of our IMUDiffusion model as some kind of early stopping criteria (OT-variants). This allowed us to reduce the amount of training epochs. Figure 6a visualizes the amount of training epochs until the training process has been terminated by this early stopping criteria. It is shown separately for each participant and each activity that the participants performed. In this graph, we only visualized the results that we obtained by using the C-Opt GAK similarity score calculated between the PSDs of the signals. A summary across all three methods is visualized in Figure 6b. The results are further divided by the four activities. In general, we can see a reduction in the amount of training epochs independent of the similarity score function used. With 1, 100 training epochs, the fastest termination of the training occurred whilst monitoring the training process using the Cosine similarity metric applied to the signals in the time domain. On average, this method required the least amount of training epochs until it stopped the training. With it, we were able to reduce the amount of training epochs by 28.70%. By using the Cosine similarity between the PSDs of the signals as metric reduced the amount of training epochs by 21.62%. Finally, the C-Opt GAK similarity metric allowed us to reduce the amount training epochs the least with a reduction of 19.51%. A summary of the reduced amount of training epochs per class and similarity metric is given in Table 2.

**Table 2:** The average training epoch at which the similarity score reached a local optima and led to an early stopping of the training of the IMUDiffusion model.

**Similarity score**	**Walking**	**Running**	**Jump up**	**Cycling**
Cosine (PSD)	3857.33 ± 579.09	3315.67 ± 820.65	3219.83 ± 740.06	3715.67 ± 615.20
Cosine (time)	3657.33 ± 682.16	2924.00 ± 860.35	3144.83 ± 871.41	3107.33 ± 883.37
C-Opt GAK (PSD)	3507.33 ± 664.84	3744.83 ± 426.96	3861.50 ± 659.58	3457.33 ± 696.97

Without this early stopping criteria, the amount of training epochs was set to 4, 500.

#### 3.1.1 Monitoring the DDPM training process of the climbing data

**Figure 7:**
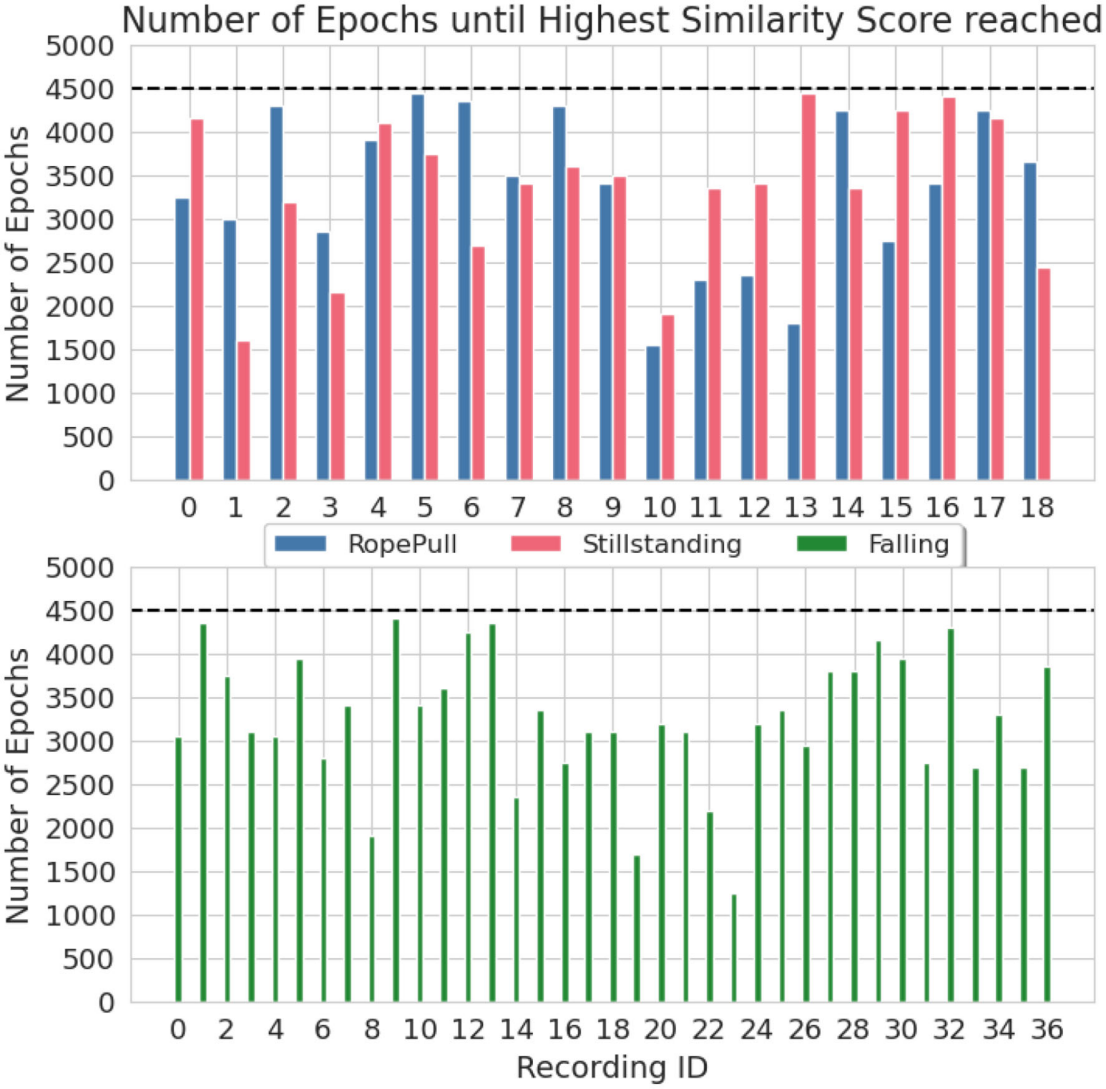
Number of training epochs until the similarity score induced an early stopping of the training process. Visualized are the recording and class individual result from the climbing dataset when using the C-Opt GAK similarity metric for early stopping. The dashed black line represents the pre-set amount of training epochs.

The C-Opt GAK similarity score was used in the training process of the DDPM to analyse the quality of the synthetic data. It allowed an earlier stop of the training before the pre-set amount of epochs have been reached. It was set to 4,500 which is identical to the HAR dataset. On average, we were able to reduce the amount of training epochs by 28% for the Falling class, 26% for the Rope-Pull class and 25% for the Stillstanding class. The amount of required training epochs per recording is visualized in Figure 7. In the best case, it allowed us to abort the training almost 3, 000 epochs earlier which translates to a reduced training time of almost 66%.

### 3.2 Monitoring the denoising process

**Figure 8:**
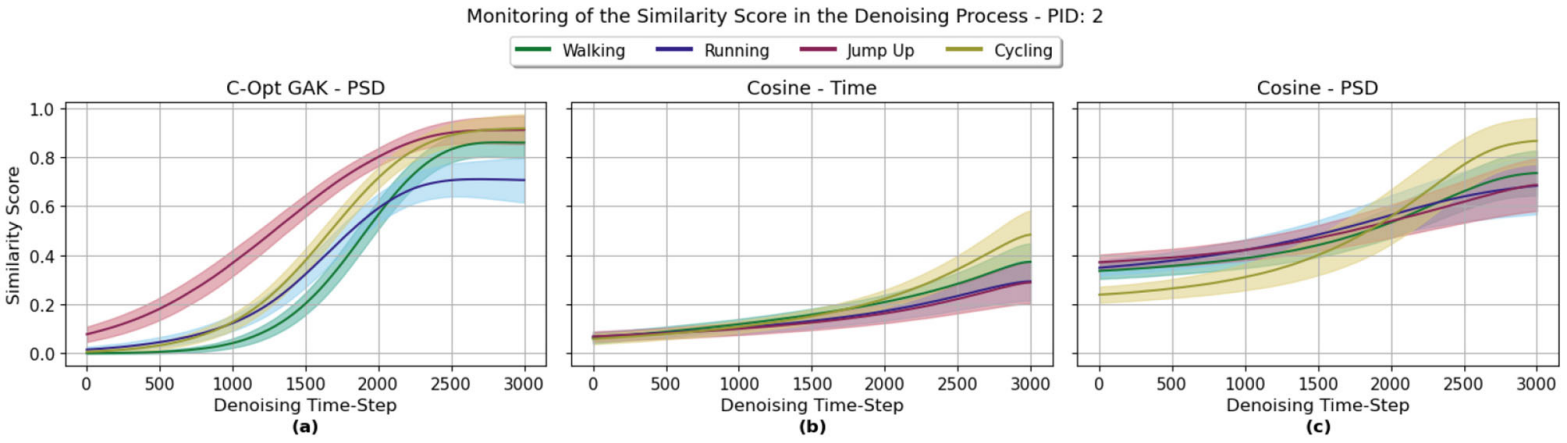
This graph visualizes the similarity scores for specific denoising steps in the denoising process for a single participant (PID 2), separated by the four activities Walking, Running, Jump Up and Cycling. **(a)** C-Opt GAK score value. **(b)** Cosine similarity score between the signals in the time domain. **(c)** Cosine similarity score once between the signals PSDs.

In this section, we describe the results from using the similarity score functions for monitoring the denoising process. An exemplary result of a monitored denoising process over one selected participant for all three metrics—C-Opt GAK, Cosine PSD and Cosine Time—is visualized in Figure 8. At the first denoising step (Denoising Time Step = 0), we start with a standard normal distributed signal. Interestingly, the Cosine similarity between the PSDs of the gaussian white noise and real sequences showed some kind of similarity, see Figure 8c. Even score values of around 0.4 were reached. The score value did still increase with the denoising steps, though, in some cases only marginally from 0.4 to around 0.6. This small increase of the score value could also be observed when the Cosine similarity was calculated between signals in the time domain, see Figure 8b. Though, this time, the score value started on average at around 0.064 and did end with a similarity score value of 0.484 on average at the last denoising step. This was at least the case for the Cycling class. For the Jump Up class, the similarity score did on average reach a value of around 0.290. Finally, the C-Opt GAK metric was able to broaden the range, see Figure 8a. For example, on average a score of around 0.0070 was reached at the first denoising step with the Cycling class. It did increase on average to 0.917.

Using the C-Opt GAK metric, highest score values were reached around the 2, 798^*th*^ denoising step on average. When using the Cosine PSD and Cosine Time metrics, highest scores were reached around the 2, 885^*th*^ and 2, 933^*th*^ denoising step respectively.

### 3.3 Classification results

**Figure 9:**
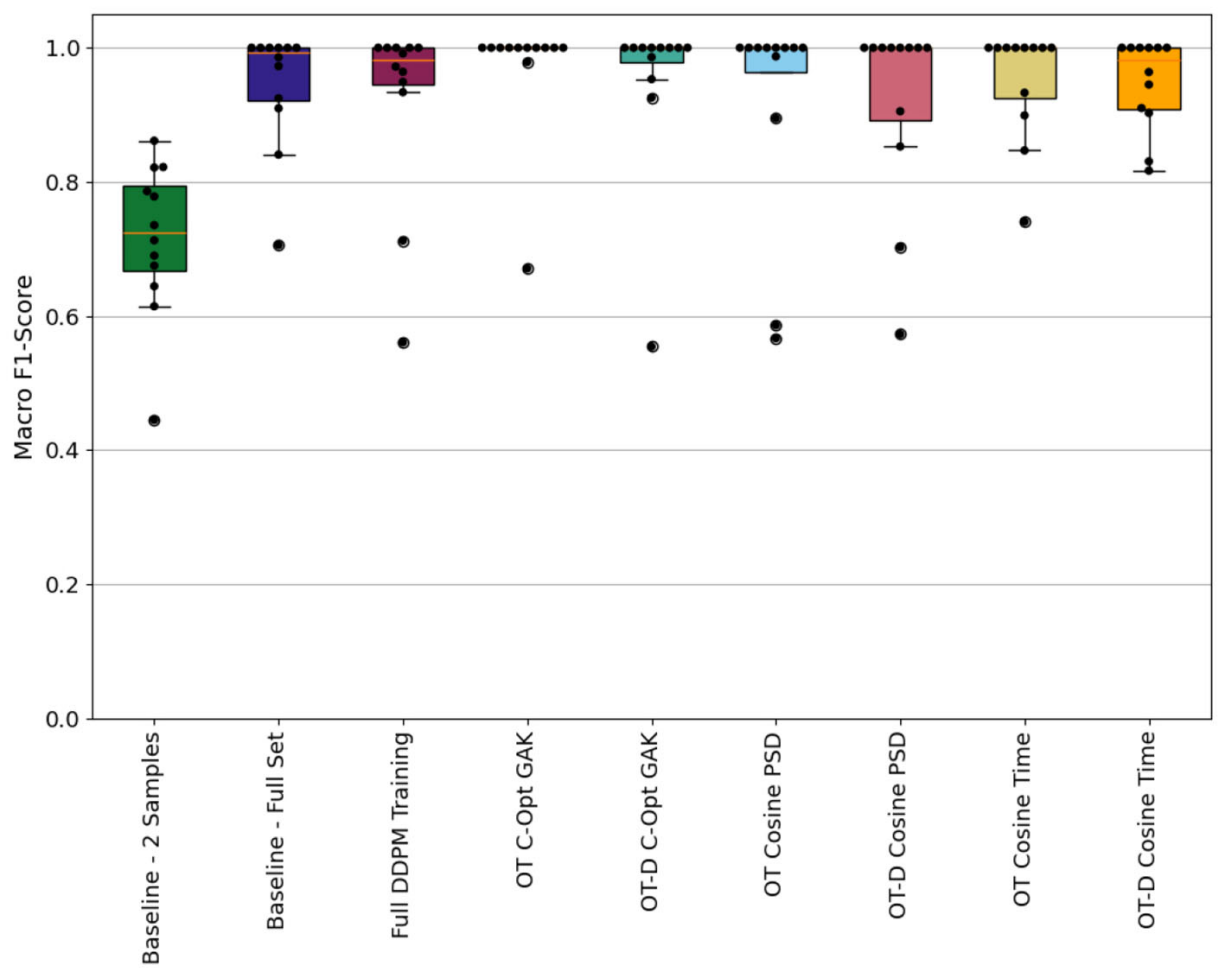
Participant individual macro F1-scores calculated with the nine classification models represented as swarm-boxplot where each dot represents one participant. Compared are the three results from the baseline models 2 Samples, Full-Set and Full DDPM Training against the results from the six models that were trained with synthetic sequences. Those sequences were generated using the similarity metrics (OT, Optimal Control Training; D, Denoising).

The results with the baseline sets have already been discussed in detail in Oppel and Munz (2025). For comparison reasons, they are still added to the evaluation and graph that visualizes the classification results, see Figure 9. To be more specific, the graph visualizes the macro F1-scores across all test subjects according to the LOSOCV approach, and that individually for all 9 training sets used to train the classifier. The macro F1-scores are visualized as a swarm-box-plot, where each dot represents the score of one left out participant and each box depicts a statistical analysis over all those subject-individual results. Best results were achieved with the OT C-Opt GAK set as with only two participants (PID 3 and 12) a macro F1-score of < 1.0 were reached. With the remaining sets we achieved higher test scores for those two participants. Except with the 2 Sample set, where the test score dropped even further. Sequences from the Running and Walking activities were mixed up with each which led to the deterioration of the score value. For PID 3, sequences from the Cycling class were also mixed up with sequences from the Jump Up class.

#### 3.3.1 Impact of using the similarity scores for early stopping in the denoising process of the DDPM

This section analyses the results of using sequences for the classification task that were generated using an early stopping criteria within the denoising process according to Section 3.2. Compared with the results obtained by using the two baseline sets 2 Samples and Full-Set for training the classifier, the early stopping of the denoising process led to sequences that mostly improved the classification task. By using the C-Opt GAK set, the macro F1-score increased for all 12 participants when compared to the results of the 2 Sample set. Against the results obtained by using the Full-Set, the macro F1-score improved for 4 participants and decreased for the participants 3 and 12. Using the Cosine metric either in the time domain or by using the PSDs of the signals, the macro F1-scores improved for 4 participants. In the same way did the score value decrease with 4, respectively 3 participants.

Visually comparing the results obtained with the OT-D sets against the OT sets when the same metric was used for early stopping showed a decreasing performance for all sets evaluated, see Figure 9. Using the C-Opt GAK metric for monitoring indeed decreases the results for the participants 1 and 16. In percentages, the scores decrease by 44.55% and 7.43% respectively. Though, it also increases for the participants 3 and 12 by 28.22% and 0.73% respectively. Analyzing the results that have been obtained using the Cosine metric, the additional early stopping of the denoising process improved the results for a single participant when the PSDs of the signals has been compared. It decreased for three participants. In the time domain, the OT-D Cosine Time set improved the results of the classifier for three participants and decreased it for four articipants.

#### 3.3.2 Impact of calculating the similarity score in the time domain or between power spectral densities

We have used the information of the Cosine similarity score for stopping the training of a diffusion model and its denoising process earlier than scheduled. The similarity itself was calculated between signals in the time domain and their power spectral densities. In this section, the classification results between those two approaches are presented. When using the similarity score only for monitoring the training process, the OT Cosine PSD set led to an improvement of the macro F1-score for two participants compared to the results obtained with the OT Cosine Time set. The scores improved once by 10.1% and once by 15.3%. In contrast to that, the classification results improved for four participants when trained with the OT-Cosine Time set. In the best case, the macro F1-score increased by 36.6% for PID 1 and the least improvement was achieved for PID 4 with an improvement of around 1.3%. When using the similarity score also in the denoising process, we achieved a higher macro F1-score for four participants with an increase of up to 42.7%, whereas for three participants we achieved a lower score value with a reduction of < 10%.

#### 3.3.3 Impact of the standard deviation range to estimate the scaling factor

**Figure 10:**
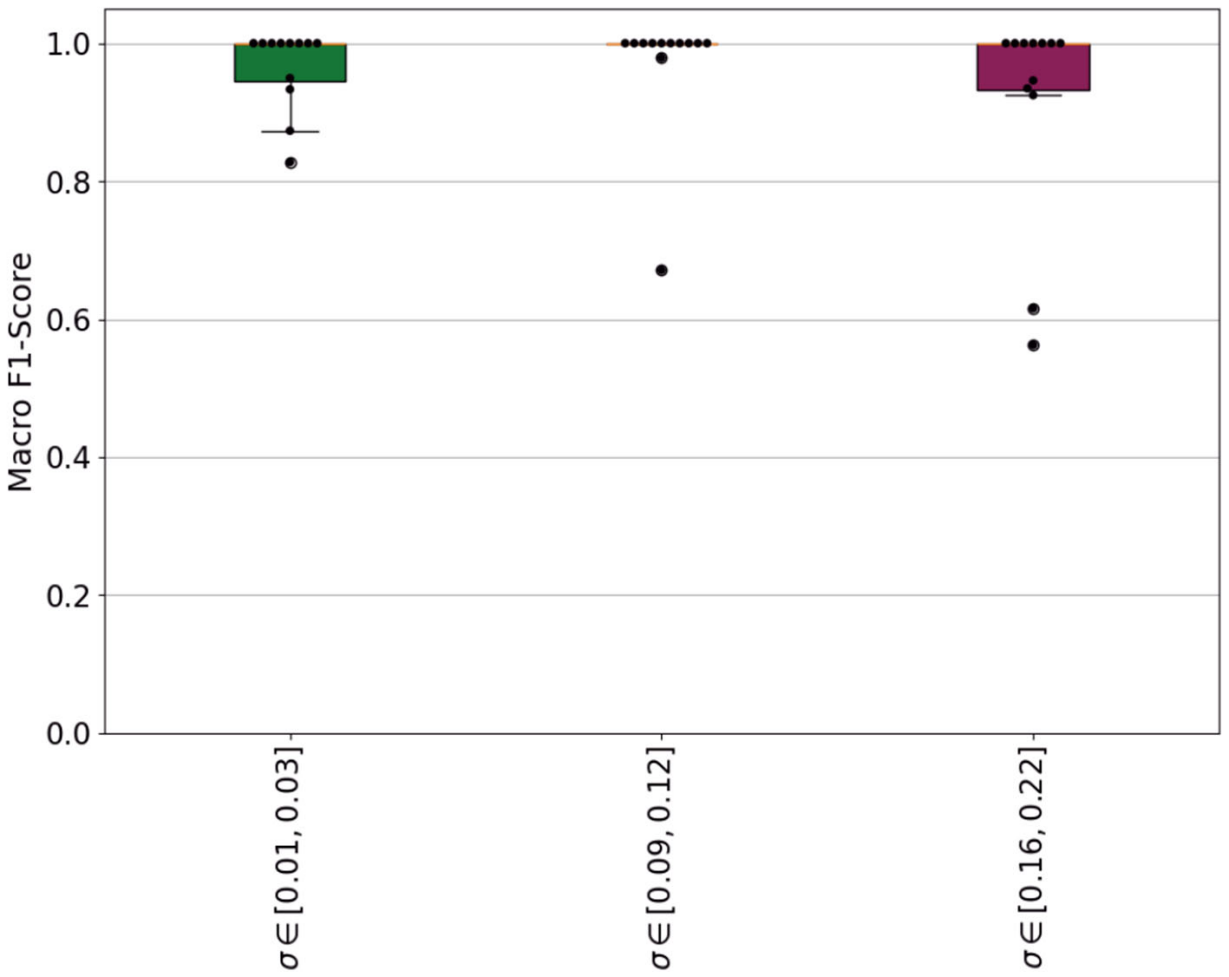
Box plot comparing macro F1-scores across three groups with standard deviations (σ) of [0.01, 0.03], [0.09, 0.12], and [0.16, 0.22]. The y-axis ranges from 0.0 to 1.0, showing variations and outliers in scores among groups. LOSOCV classification results using the macro F1-score as evaluation metric. Three swarm-boxplots are depicted; one for each optimization criteria which was used to find an optimal scaling factor. Each dot represents one participant which was held out during the training of the classifier.

In the following, we analyze the pre-defined optimization constraint to find the optimal scaling factor σ, namely the range of standard deviation. For this, we compared the chosen interval to two varying ranges: a lower and a higher range. We chose those intervals as follows: ${\hat{\sigma }}_{\vartheta_{1}}=\left[0.01,0.03\right]$ and ${\hat{\sigma }}_{\vartheta_{3}}=\left[0.18,0.22\right]$. This results into different scaling factors and, ultimately, a different similarity value between two sequences. We used those values to monitor the training of the DDPM as an early stopping criteria and then generated synthetic sequences from those models. The synthetic sequences were then used to train a classifier. The results are visualized in Figure 10. The boxplots depict the macro F1-scores for each participant individually. The optimization criteria ${\hat{\sigma }}_{\vartheta_{2}}$, which was chosen based on a preliminary data analysis, lead to only two score values of less than 1.0. The other two ranges lead to score values of less than 1.0 in at least twice as many participants.

#### 3.3.4 Evaluation of the classification task with the climbing dataset

The results are summarized in Table 3. We compare two different evaluation approaches. The first one calculates the Geometric Mean for each climbing dataset between all three classes: Falling, Rope Pull and Stillstanding. The second approach addresses the models capability of differentiating between a fall and an ascend in general by combining the Rope Pull and Stillstanding class. This second approach is relevant if the model has to predict a fall in real time. Therefore, it is not relevant whether the sequence from the ascend belongs to a Rope-Pull or not.

**Table 3:** Classification results from the different climbing datasets with the geometric mean as evaluation metric.

**Evaluation metric**	**TRTR**	**TRTR and downsampling**	**TRTR and oversampling**	**Best step TSTR**	**Last step TSTR**
3 class geometric mean	0.420 ± 0.210	0.858 ± 0.029	0.857 ± 0.033	0.874 ± 0.045	0.892 ± 0.030
Fall vs. climbing geometric mean	0.326 ± 0.163	0.927 ± 0.027	0.895 ± 0.039	0.930 ± 0.052	0.931 ± 0.031

The TSTR models were trained with 1,000 synthetic sequences.

The results obtained by using the TRTR model did reach an average Geometric Mean of 0.420 and it was not able to clearly separate fall sequences from ascending sequences. In one of the five cross-validation steps, none of the fall sequences were correctly classified. By balancing out the class imbalance of the training set, the metrics increased for both approaches. Both downsampling the majority classes as well as oversampling the minority class improved the classification results for both approaches. Using synthetic sequences for training the classifier instead of only real sequences improved the metrics in all evaluated areas as shown in Table 3. Highest metric values were achieved by utilizing the synthetic sequences obtained from the Last Step DDPM model. Using the sequences from the Best Step model decreased the metrics by 1.5% for the 3 class and by 0.1% for the 2 class evaluation approach. The reduced score value for the 3 class evaluation results from the prediction of the Rope Pull class as its sensitivity value dropped from 85.55% to 80.64%.

## 4 Conclusion

To rely on an objective criteria to supervise the training of a neural network classification model is a normal approach to stop the optimization process at the most beneficial timestep. In contrast, the supervision of time series DDPMs is not as straightforward and mostly based on the knowledge of the user. An objective criterion is missing in this field. We tried to fill this gap by integrating existing and novel similarity score functions into the training and denoising process of a time series DDPM. The novel similarity score function is based on an existing alignment function which we adapted to best fit the underlying dataset. Therefore, we fit the similarity function to the training and validation sets by adjusting the scaling factor σ of the initial similarity score function GAK. The idea is to find an optimal σ to assert high similarities between signals under the assumption that dissimilarities still exist within the sequences. The adapted similarity score function is called C-Opt GAK. The similarity metrics can then be used to not only monitor the diffusion models performance, but also for stopping the training and denoising as soon as an optimal similarity between real and synthetic sequences was achieved. Generated sequences then have been used to train a classifier with the task of differentiating the four classes. It served as an objective criteria for evaluating the effectiveness of using similarity scores to optimize the training and synthetization process of diffusion models.

By using the C-Opt GAK metric for early stopping the training of the diffusion model, we were able to reduce the amount training epochs on average by 20%. Across all participants and classes, this saved us 41, 148 epochs. This not only saved computation time, but the classification results improved with six participants compared to when the classifier was trained with synthetic sequences that were generated after training the diffusion model for the full 4, 500 epochs. For one participant, the macro F1-score increased by up to 43.9%. Independent of the similarity metric used for early stopping, the macro F1-score increased with more participants as it decreased, showing the effectiveness of this approach.

Another approach is to integrate the similarity metrics in the denoising process. This allows to stop the process as soon as highest similarity between sequences was reached. Depending on the similarity metric, the score value itself could be misleading. The score values from the Cosine similarity between power spectral densities showed similarity values between 0.2 and 0.4 when real sequences have been compared against signals depicting random gaussian noise. Nevertheless, the classification results improved with five participants compared to the results obtained with the Full DDPM set. In contrast to that, the results dropped for either two or three participants depending on the similarity metric used.

In addition to the HAR dataset which contains cyclic movements, we extended the analysis on another acyclic time series dataset out of the domain of sport climbing. We could show that integrating our C-Opt GAK metric in the training process of a generative diffusion model reduced the amount of necessary training epochs significantly by over 25% for this dataset. Though, it lead to a slight reduction of the 3 class classification performance but maintained the same classification performance on the 2 class problem.

## 5 Discussion and outlook

In this paper, we investigated the possibility of using similarity score functions to monitor the training and denoising process of a DDPM. The effectivity of those score functions was shown on a real world human activity recognition dataset. We were able to reduce the amount of training epochs as well as denoising steps without missing out on the key characteristics that define the human activity it represents. This could be verified by using those generated sequences to train a classifier. For most LOSOCV steps, the additional synthetic sequences which were generated with the monitored DDPM improved the separability of the classifier. Even though this was not the case with all participants. With the help of the similarity score we were able to estimate the quality of the synthetic sequences, which resulted in the identification of sequences showing high dissimilarities. It would be wise to integrate a selection process to identify the most suitable sequences improving the classifiers performance even further and reduce the required amount of synthetic sequences to a minimum.

The monitoring of the denoising process including an early stopping criterion is a non-intuitive approach. The diffusion model was trained in combination with a pre-chosen scheduler, which is in the generation process responsible for removing noise successively. So, stopping the denoising process earlier is leading to sequences containing more shares of high frequency noise compared to sequences generated after the last denoising step. We were still able to maintain the quality of the classifiers separability compared to the classifiers trained with the baseline training sets. Though, it might affect different time series signals from other sensor types or other activities from the same sensor type differently. So, it would be recommended to test this approach for different types of sensors and activities. The advantage of requiring less denoising steps in the synthetization process is unambiguous. It reduces the time to generate the sequences. Additionally, it would be interesting to test different methods that reduce the amount of denoising steps for a DDPM against this approach.

The range of the standard deviation for the calculation of the σ-value for the C-Opt GAK similarity score was chosen based on subjective criteria based on a preliminary data analysis. We compared it against two different optimization constraints to consolidate the choice of our subjective one. Yet, this preliminary analysis beforehand is a time consuming process and it would be desirable to find an automated process to identify the optimal range.

The climbing dataset was used to extend the usage of the similarity metrics in the training process of a generative diffusion model to an acyclic time series dataset. Our findings are promising, as we were able to reduce the required amount of training epochs significantly whilst also increasing the performance of an underlying classification task against the baseline models. Still, stopping the training process of a diffusion model too early can have a negative impact on the quality of the generated data. This could be seen in the performance drop of our classifier. Another explanation for this drop might also lie in the randomness of the data generation process. We used 1, 000 randomly chosen synthetic sequences to train each of the two TSTR models individually and as the C-Opt GAK similarity score shows exemplarily in Figures 4a, b, the diffusion model seems to have generated sequences which are fairly dissimilar to the real sequences. As those are in part responsible on the separability of the classifier, it would be interesting to elaborate further on the relation between the similarity score, the quantity and quality of the synthetic sequences and their impact on the classification task.

## Data Availability

Publicly available datasets were analyzed in this study. The HAR dataset can be found at: https://archive.ics.uci.edu/dataset/305/realdisp+activity+recognition+dataset; REALDISP Activity Recognition Dataset.
